# Insertion of Ahmed Implant With Partial Buckle Resection: A Case Report

**DOI:** 10.7759/cureus.31453

**Published:** 2022-11-13

**Authors:** Asuka Noguchi, Shunsuke Nakakura, Santaro Noguchi

**Affiliations:** 1 Ophthalmology, Saneikai Tsukazaki Hospital, Himeji, JPN

**Keywords:** ahmed implant, trauma, retinal detachment, encircling, buckling, glaucoma

## Abstract

This report describes a case of poor intraocular pressure control after the encircling procedure for traumatic retinal detachment. We inserted an Ahmed Glaucoma Valve Implant® (AGVI) with partial sponge resection and obtained good results. The results are reported here.

An 11-year-old boy had a traumatic globe rupture in the right eye (OD). Corneo-scleral repair and lens extraction were performed on the injured eye. About one month after the injury, the intraocular pressure (IOP) of OD had increased to 25 mmHg. Glaucoma eye drops were started, and the IOP was subsequently controlled at 11-19 mmHg. Five months after the injury, the total retinal detachment was observed, and the encircling procedure with a silicone sponge was performed. Soon, right IOP control deteriorated, increasing to over 30 mmHg despite the maximum eye drops dosage. Given the poor condition of the cornea and iris after the trauma and the limited surgical space after the encircling procedure, we chose to partially cut the sponge and insert the AGVI.

Intraoperatively, the adhesions between the conjunctiva and the Tenon’s capsule were dissected. The sponge was partially cut at the 10 o’clock position, and both ends were sutured to the sclera. The AGVI was subsequently inserted into the space obtained. The plate was placed posterior to the sponge, and the tube was placed between the cut sponges and inserted into the anterior chamber. The right IOP was 8 mmHg on the day after the surgery and remained at 15-20 mmHg until nine months after surgery postoperatively under two medications. No recurrence of retinal detachment was further observed. In our case of post-traumatic glaucoma, the partial removal of the sponge along with the insertion of an AGVI has shown beneficial results in terms of IOP control.

## Introduction

Ocular trauma is one of the leading causes of blindness worldwide [1,2]. It can be classified into open-globe injury and closed-globe injury, but in both cases, traumatic retinal detachment or traumatic glaucoma may occur after the injury, and both may be complicated. In cases of lens injury or vitreous hemorrhage, traumatic glaucoma often follows [3-7]. In addition, elevated intraocular pressure (IOP) often occurs after vitreoretinal surgery or scleral buckling surgery [8-10]. Glaucoma surgery after scleral buckling surgery for retinal detachment is difficult, and trabeculectomy may result in poor postoperative outcomes in patients with conjunctival scarring. Furthermore, the presence of the buckle may limit the operative space and cause problems during implant insertion. In this report, we describe good outcomes from inserting an Ahmed Glaucoma Valve Implant® (AGVI) using partial buckle resection after the encircling procedure for traumatic retinal detachment.

## Case presentation

**Figure 1:**
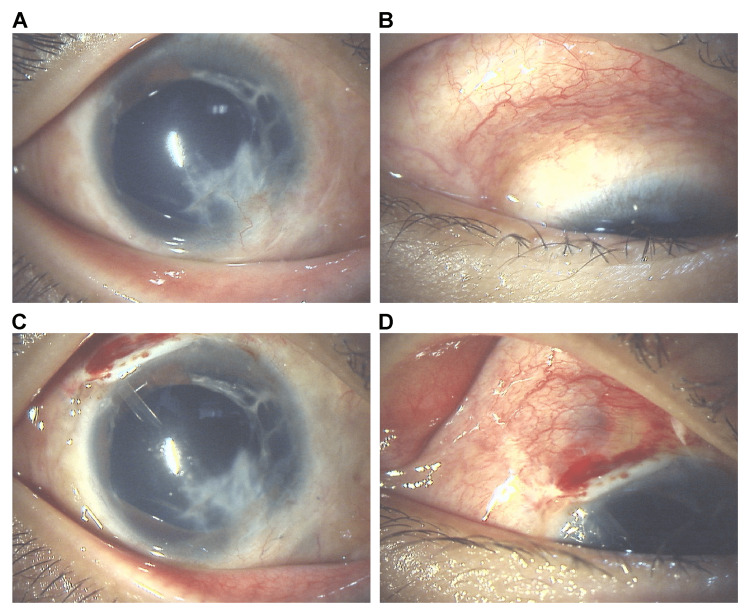
Preoperative and postoperative anterior segment photograph A: Preoperative anterior segment photograph showing strong corneal opacity and conjunctival invasion in the 1–6 o'clock direction and iris loss in the 1–4 o'clock direction. B: Preoperative anterior ocular photograph of the superior temporal region. C: Postoperative anterior segment view showing a tube inserted into the anterior chamber at 10–11 o'clock. D: Postoperative anterior ocular view of the superior temporal region. The sponge in the area indicated by the arrow has been removed and a tube has been placed in the same area.

An 11-year-old boy had a traumatic globe rupture in the right eye (OD) when he struck it with a stick while playing. He was injured in March 2020. The eye ruptured due to blunt perforation of the sclera and iris. The choroid and part of the vitreous were prolapsed, and a hematoma formed in the anterior chamber. On the same day, the patient underwent sclerocorneal sutures and lens extraction under general anesthesia. About one month after the injury, the right IOP increased to 25 mmHg. Glaucoma eye drops were started, and the right IOP was maintained at 11 to 19 mmHg. About five months after the injury, OD developed proliferative vitreoretinopathy due to total retinal detachment of unknown origin. During this period, he was followed up every month, with a fundus examination each time. Encircling surgery was performed using a silicone sponge for retinal detachment. Preoperative right visual acuity (RV) was 0.01 and postoperative RV was c.f/50cm. One month after the surgery, the right IOP became poorly controlled and did not respond to an additional dose of eye drops. The right IOP reached over 30 mmHg despite medication (latanoprost 0.005%, a combination of dorzolamide hydrochloride 1% and timolol maleate 0.5%, and ripasudil hydrochloride hydrate 0.4%). The gonioscopic examination showed that the 1/2 quadrant was open and the 1/2 quadrant was not visible due to corneal opacity. Because of the poor post-trauma condition of the iris and cornea and the limited surgical field after the encircling procedure (Figures 1A, 1B), we decided to partially remove the sponge and insert an AGVI after the encircling procedure. The detailed surgical procedure is described below. Preoperative (Figures 1A, 1B) and postoperative (Figures 1C, 1D) anterior segment photographs are shown in Figure 1.

IOP reduction was achieved postoperatively, and the right IOP was 8 mmHg without medication on the day after surgery. Due to high postoperative IOP, latanoprost 0.005% and dorzolamide hydrochloride/timolol maleate were administered, but latanoprost 0.005% was stopped three months after the surgery because the right IOP was stable and the right IOP was 17 mmHg at that time. When the patient was examined one month later, no increase in IOP was observed. Since then, the patient has been taking dorzolamide hydrochloride and timolol maleate solution, and his right IOP has remained at 15-20 mmHg until nine months after surgery, and RV was c.f./40cm at this point. In addition, fundus examinations were performed at every visit, and no recurrence of retinal detachment or other postoperative complications were observed. The postoperative anterior segment photographs are shown in Figures 1C, 1D.

Surgical procedure

**Figure 2:**
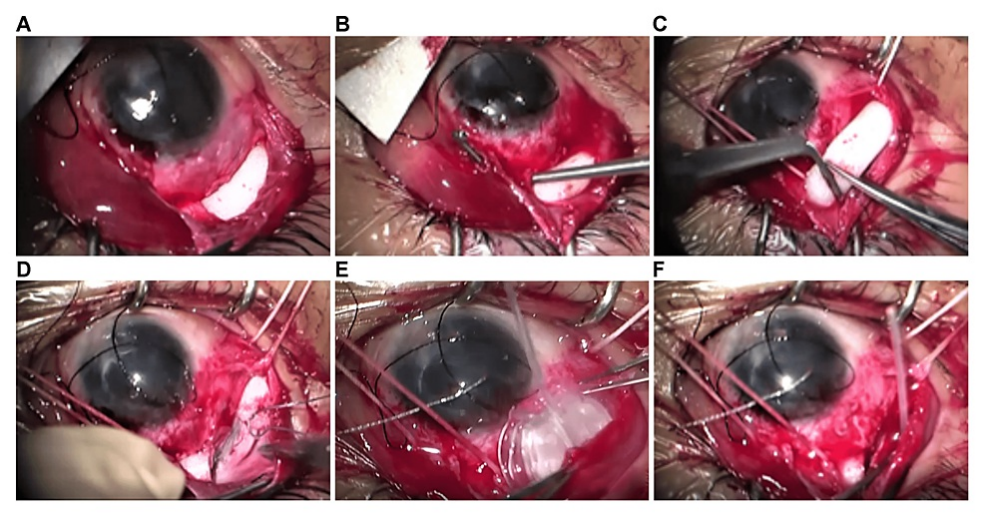
Photographs during surgery All photos were taken during surgery. A: The conjunctiva was incised to expose the sponge. The distance from the corneal limbus to the proximal end of the sponge was approximately 5 mm. B: A fornix-based flap of conjunctiva and Tenon capsule was raised in the quadrant at 9 to 12 o'clock, and the external and superior recti muscles were identified. C: Picture taken just before cutting the sponge in the 11 o'clock direction. The cut end of the sponge was firmly grasped and sutured to the sclera immediately after cutting. The sponge was cut and sutured in the same way in the 10 o'clock direction. D: The 10–11 o'clock sponge was cut and the end was fixed to the sclera with 5-0 Dacron. E: The Ahmed Glaucoma Valve Implant® (AGVI) was inserted into the deeper position. F: A tube was inserted into the anterior chamber. The tube passed through the excised part of the sponge and the plate was implanted behind it.

The surgery was performed by two doctors: one who did the encircling procedure and the other who was in charge of treating glaucoma. General anesthesia was administered using subconjunctival and sub-Tenon anesthesia via 2% xylocaine injection with epinephrine in the operative field. A 5-0 silk was threaded through the corneal ring at the 11 o’clock position. A fornix-based flap of the conjunctiva and Tenon’s capsule was raised in the 9-to-12-o’clock quadrant. The thick Tenon’s capsule was traction sutured in the 10 and 1 o’clock directions using 5-0 silk (Figure 2A). The conjunctiva and Tenon’s capsule were dissected until the silicone sponge, lateral rectus muscle, and superior rectus muscle. The control thread was applied to each ocular muscle (Figure 2B). A part of the sponge at the planned insertion point (10 to 11 o’clock) was removed (Figure 2C), and the end of the sponge was sutured to the sclera using 5-0 Dacron to prevent recurrence of retinal detachment due to the loosening of the buckle (Figure 2D). After confirming that water could pass through the AGVI (model FP7), the plate was inserted into the upper temporal area, and the tube was placed between the excised sponges (Figure 2E) and inserted into the anterior chamber after puncturing with a 23G needle at 1.5 mm from the corneal limbus (Figure 2F).

We did not fix the plate. The tube was fixed with 8-0 Vicryl and covered with the preserved cornea and fixed with 8-0 Vicryl. Conjunctival sutures were performed with 8-0 Vicryl, IOP was adjusted, and the surgery was completed.

## Discussion

Two main treatment methods are applied for retinal detachment: vitreoretinal surgery and scleral buckling surgery. Scleral buckling surgery is mainly chosen for younger patients, and, in the case of intractable retinal detachment, vitreoretinal surgery may be combined with encircling surgery. Tires and sponges are the options for implants used in scleral buckling surgery. Many different types with different thicknesses and shapes can be used, depending on the type of case and the surgeon’s preference. If the buckling is applied only to some quadrants, glaucoma surgery can be performed in the vacant quadrants. If a silicone tire or band is used for the buckling surgery, the implant can be inserted through the tube over the buckle because of its lesser thickness.^ ^

Scott et al. [11]^ ^reported the insertion of a Baerveldt device in 16 eyes after scleral buckling. They implanted the Baerveldt over or behind the encircling band and over the adjacent recti muscles. They selected for Baerveldt implantation the quadrant with the least amount of episcleral hardware from previous retinal surgery (i.e., if a segmental or radial scleral buckle rather than an encircling element was present, then a quadrant without the buckle was selected as the surgical site for implantation). This method has the advantage of allowing the surgeon to insert the implant in the usual procedure, but it may be difficult when the operative field is limited after encircling with a sponge, as in our case.

Sidoti et al. [12] reported a series of 13 patients in whom a silicone tube was implanted to shunt the aqueous humor from the anterior chamber to the fibrous capsule surrounding a silicone scleral-encircling element placed during a prior operation for retinal detachment repair. A silicone tube was inserted through a small incision into the fibrous capsule overlying the scleral implant. Since the distal end of the tube is already inside the formed membrane, it offers a low risk of postoperative hypotony without ligation of the tube. However, four of the 13 cases (30.8%) required reoperation due to fibrous occlusion of the tube, and one of them required removal of the tube and reinsertion of a Molteno implant because of repeated tube occlusion.

Smith et al. [13] inserted drainage tubes in 11 eyes after scleral buckling. In seven of the 11 cases, a long valved Krupin Denver tube was passed through the capsule around the silicone tire using a method similar to that of Sidoti et al. In two of these seven cases (28.6%), fibrous obstruction of the distal portion of the tube occurred, thus requiring reoperation. Other complications, such as leakage from the conjunctiva and abnormal tube positioning, occurred, and reoperation was not necessary in only one of the seven cases. In the remaining four cases, the Baerveldt implant was trimmed to a smaller size and fixed under an encircling band, and the tube tip was inserted into the anterior chamber. Although the risk of tube obstruction is low with this method, the risk of scleral perforation and eye rupture is a concern because of the additional pressure applied to the thinning sclera after buckling [14].

Suh et al. [15] performed the same method of passing the distal part of the tube into the capsule around the encircling band in seven eyes of five patients. Postoperative examination showed no tube occlusion, and the only complication was tube misalignment in two eyes. Similar to Sidoti et al. and Smith et al., this method offers advantages, such as early postoperative IOP reduction, a relatively easy technique, and a low risk of postoperative hypotony. However, the buckling material must be thin, narrow, and located posteriorly.

When a sponge is used as the material for the buckling surgery, as in the present case, the volume of the buckle is large; thus, implanting a tube over the buckle would increase the area and pressure of the tube in contact with the conjunctiva, which would increase the risk of postoperative complications. We also considered the option of passing a tube under the sponge, but the tube would be more liable to compression, and the IOP-lowering effect would be insufficient. Therefore, we chose to partially remove the sponge in the present case.

Normally, the superior or inferior temporal quadrant is chosen as the implant insertion site because it is relatively less likely to cause complications. In the present case, we chose the superior temporal quadrant because the inferior nasal side sustained the most damage from the trauma, which resulted in poor intraocular visibility due to strong corneal opacity. Furthermore, the inferior nasal side sustained the worst retinal detachment. For this reason, we thought inserting the implant as far away from the inferior nasal side as possible was ideal.

Usually, the plate is fixed to the sclera with 8-0 Vicryl before inserting the tube into the anterior chamber. However, in our case, we did not fix the plate because we were able to confirm that the plate did not come forward since both ends of the sponge acted as a dike and did not shift to the back, the sclera was quite thin, and the large-volume sponge made it difficult to thread the plate and sclera. Nevertheless, fixing the plate to the sponge instead of the sclera would have been optimal.

## Conclusions

Glaucoma surgery after scleral buckling surgery for retinal detachment is difficult, and the presence of the buckle may limit the operative space and cause problems during implant insertion or other types of glaucoma surgeries. In our case of post-traumatic glaucoma, the partial removal of the sponge along with the insertion of an AGVI has shown beneficial results in terms of IOP control.
